# Accountability, ambition, and quantifiable action in the carbon emission reduction plans of the ten largest pharmaceutical companies in Australia: a cross‐sectional analysis

**DOI:** 10.5694/mja2.52621

**Published:** 2025-03-04

**Authors:** Hayden Burch, Georgia Brown, Oliver Adler, Jason Wong, Kenneth D Winkel

**Affiliations:** ^1^ Melbourne Medical School the University of Melbourne Melbourne VIC; ^2^ Austin Health Melbourne VIC; ^3^ New England Local Health District Newcastle NSW; ^4^ Melbourne School of Population and Global Health the University of Melbourne Melbourne VIC

**Keywords:** Climate change, Health policy, Health systems, Pharmacy

## Abstract

**Objectives:**

To assess the commitment of the ten largest pharmaceutical companies operating in Australia to achieving net zero emissions by evaluating their accountability metrics, ambitions, and quantifiable actions taken.

**Study design:**

Cross‐sectional study; analysis of publicly available company reports published during 12 December 2015 – 31 December 2023.

**Setting, participants:**

Ten largest pharmaceutical companies operating in Australia, defined by total pharmaceutical costs (to patients and Pharmaceutical Benefits Scheme) for PBS‐subsidised medications, as reported in PBS expenditure and prescriptions reports for 2020–21 and 2022–23.

**Main outcome measures:**

Content analysis of publicly available documents for the ten companies using modified criteria from the PricewaterhouseCoopers *Building blocks for net zero transformation framework*, with three domains: accountability, ambition, and action; the Carbon Disclosure Project (CDP) grading; the Science Based Targets initiative (SBTi) approval system. We focused on measurement, target setting, and achievement of emission reductions, and ranked the environmental sustainability of companies using a points and colour coding system.

**Results:**

Three groups could be defined by evidence of their commitment to emissions reductions. The first — companies leading emissions reduction efforts, with SBTi‐approved near term targets, consistent emissions monitoring, well defined commitments, and quantified evidence of action — includes AstraZeneca, Novartis, Johnson & Johnson, Bayer, and Merck & Co. The second group — companies that had made commitments to SBTi‐approved targets but their disclosure records are limited — includes AbbVie and Roche. The third group — without public commitments to achieving net zero emissions, minimal or no SBTi‐approved targets, and minimal disclosure or monitoring of emissions — includes Viatris, Vertex, and Arrotex.

**Conclusions:**

The ten largest pharmaceutical companies in Australia are moving towards net zero greenhouse gas emissions at different rates. Gaps in standardised reporting processes should be closed, and further qualitative research on industry‐wide environmental sustainability policy and practice is needed.

**The known**: The ramifications of climate change for health have led to calls for reductions of greenhouse gas emissions in all sectors. In Australia, the pharmaceutical industry is responsible for about 19% of greenhouse gas emissions in the health care sector.

**The new**: Our assessment of their commitment, monitoring, and actions during 2015–2023, based on publicly available documents, indicates that the ten largest pharmaceutical companies in Australia are moving to net zero emissions at different speeds.

**The implications**: Our findings can assist policy makers and clinicians make informed decisions about low carbon suppliers of medicines and support emissions reduction efforts by pharmaceutical corporations.

Pharmaceutical companies have an environmental impact in terms of waste generation and disposal, greenhouse gas emissions, air pollution, and water, plastic, and energy consumption. In Australia, the pharmaceutical industry is responsible for about 19% of greenhouse gas emissions in the health care sector,^1^ and receives about 16% of government spending on health, principally through Pharmaceutical Benefits Scheme (PBS) subsidies (2022–23: $16.7 billion).^2^

The 2015 United Nations Paris Agreement required countries to begin reporting and reducing carbon emissions.^3^ Several international protocols standardise monitoring and reporting, set science‐based emissions reduction targets, and promote accountability and measurable outcomes.^4^, ^5^, ^6^ Standards improve sustainability disclosure and complement rather than substitute for each other.^7^ Under the Greenhouse Gas Protocol, emissions are categorised as scope 1, 2, and 3 emissions (Box 1).^5^, ^8^

Box 1 **Definitions of greenhouse gas emissions by the Greenhouse Gas Protocol^5^, ^8^**

**Table:** 

Emissions scope	Definition
Scope 1	Direct emissions from sources owned or controlled by the company: for example, emissions from combustion in owned or controlled boilers, furnaces, and vehicles; chemical production in owned or controlled process equipment.
Scope 2	Indirect emissions from generation of purchased electricity consumed by the company.
Scope 3	Other indirect emissions related to activities of the company but from sources not owned or controlled by the company: for example, extraction and production of purchased goods; upstream/downstream transportation and distribution; processing and use of sold products.

In Australia, the *National Greenhouse and Energy Reporting Act 2007* stipulates thresholds that determine which corporations are required to disclose emissions to the Clean Energy Regulator.^9^ No Australian subsidiaries of pharmaceutical companies are registered under the Act.^10^ On 27 March 2024, Treasury proposed an amendment to the *Corporations Act 2001* to make climate‐related corporate financial disclosure mandatory for large organisations, including pharmaceutical companies;^11^ such an amendment would bring Australian requirements into line with those of the European Union, the United Kingdom, New Zealand, and Japan.^11^, ^12^

The parent companies of multinational companies that operate in Australia are under increasing international pressure to reduce their environmental impact. The English National Health Service (NHS), for example, will stop purchasing from companies that “do not meet the NHS commitment to net zero by 2030”,^13^ in accordance with the procurement policy of the United Kingdom government^14^ and its commitment to net zero emissions throughout its supply chains by 2045.

An Oxford University team has recently provided the most robust assessment of the strategies of the twenty largest pharmaceutical companies for achieving net zero emissions.^15^ The authors examined 2020–21 data, and noted that nineteen companies had committed to reducing emissions, primarily by reducing scope 1 and 2 emissions, with less consistent aims for scope 3 emissions. The Oxford study was largely descriptive, and the authors recommended further comparative analyses and exploring differences by country and region.^15^

We therefore assessed the commitment of the largest pharmaceutical companies operating in Australia to achieving net zero emissions by evaluating their accountability metrics, ambition, and quantifiable actions taken. Our comparative analysis aims to inform decision making by Australian clinicians, health service leaders, and policy makers.

## Methods

We undertook a cross‐sectional analysis of publicly available documents for the ten largest pharmaceutical companies operating in Australia during 12 December 2015 (date of the Paris Agreement) to 31 December 2023. We report our analysis according to the STROBE guidelines.^16^

The ten largest companies were defined as those associated with the highest total pharmaceutical costs (ie, combined costs to patients and the PBS) for PBS‐subsidised pharmaceuticals, as reported in the PBS expenditure and prescriptions report for 1 July 2020 – 30 June 2021,^17^ and updated using the 2022–23 report.^2^ Merck Sharp & Dohme (Australia) and Vertex Pharmaceuticals (Australia) were included in our analysis because they were ranked in the top ten in 2022–23 (but not in 2020–21). We also included Roche Products (eleventh largest in 2022–23) instead of Sandoz (tenth largest in 2022–23) because Sandoz had separated from Novartis Pharmaceuticals in a 100% spin‐off in October 2023,^18^ and the available data were consequently inadequate for analysis. We excluded Efficient Funding of Chemotherapy program items, including doctors’ bag and co‐payment prescriptions, as they are not included in annual PBS expenditure and prescriptions reports.^2^

### Information sources

We assessed data in publicly available company documents — annual reports, environmental and social governance reports, supply chain communications, policy statements, investor updates, and annual financial reports — supplemented by sustainability report data, press releases, interim sustainability updates, and sustainability strategies published on the Australian websites of each company. If information was not available on the Australian website, we searched for it on the parent company website to derive a best case scenario for inclusion in the analysis. The most recent CDP submission for each company was identified by searching the CDP website. Disclosed SBTi‐approved interim and net zero emission reduction targets were validated on the SBTi website and included in the analysis.

We included pharmaceutical company documents published in English during 12 December 2015 – 31 December 2023 that included information about accountability, ambition, or action related to greenhouse gas emissions relevant to Australia for which the full text was available. We excluded superseded documents, documents primarily concerned with environmental topics other than greenhouse gas emissions, and documents otherwise deemed irrelevant to our study (eg, those discussing cultural environment). We sought data for the period 1 May 2022 – 31 March 2024 to allow for lags in reporting.

We focused on greenhouse gas emission measurement and accountability metrics, climate change targets, and quantifiable actions to reduce emissions. We extracted basic company information, and information about climate change targets, greenhouse gas emissions, and reported initiatives or strategies for reducing emissions. To check our extraction method, we also searched for “environmental sustainability”, “greenhouse gas emissions”, “carbon footprint”, “carbon neutral”, “net zero”, “energy”, “scope 1”, “scope 2”, and “scope 3”. Scope 1, 2, and 3 greenhouse gas emission data were updated for the most recent annual data available.

### Content analysis

We used three international frameworks to map the extracted information. The first comprised our modification of criteria described in the PricewaterhouseCoopers *Building blocks for net zero transformation framework*,^19^ which provides decarbonisation guidance for companies from various sectors, sizes, and locations; we selected this framework for its wide ranging and sector‐neutral approach. We modified the criteria by collapsing the nine building blocks to three domains: accountability, ambition, and action (Box 2). We then integrated the Carbon Disclosure Project (CDP) grading, a global disclosure and scoring system based on voluntary environmental impact report data published by businesses.^6^ Finally, we integrated the Science Based Targets initiative (SBTi) approval system, a global, non‐profit framework established to help companies meet industry‐specific emission reduction goals in line with the Paris Agreement.^4^ Framework adaptations were validated using face validity and compared using the methodology of the Oxford team.^15^

Box 2 **Comparative analysis of monitoring, commitment, and actions to net zero greenhouse gas emissions**

**Table:** 

Domain/subdomain	Criterion
A. Accountability: monitoring and disclosure of emissions	
A.1. Baseline disclosure	Has the company conducted a baseline assessment of its scope 1 to 3 emissions?
A.2. Progress disclosure	Does the company disclose its progress by performing annual scope 1 to 3 assessments?
A.3. Standardised disclosure	Does the company use standard external frameworks when reporting emissions?
B. Ambition: making forward‐looking commitments to emission reductions	
B.1. Science‐based target	Does the company define a science‐based net zero emissions target with near (within ten years) and long term (beyond ten years) milestones?
B.2. Mitigation hierarchy	Does the company prioritise emissions abatement over neutralisation and compensation measures?
B.3. Specific commitments	Does the company outline areas of future business activity with specific timeframes to achieve its decarbonisation strategy?
C. Action: substantiating commitments with changes to business activity	
C.1. Scope 1 and 2 action	Has the company made changes to its operations or infrastructure to reduce scope 1 and 2 emissions?
C.2. Scope 3 action	Has the company made changes to its operations or infrastructure to reduce scope 3 emissions?
C.3. Ancillary action	Is the company fostering a corporate environment congruent with its decarbonisation strategy (governance, innovation, financing, public engagement)?

### Statistical analysis

We focused on measurement, target setting, and achievement of emission reductions, and ranked the environmental sustainability of companies using a points and colour coding system (Box 3). Three authors independently scored each company; final score allocations were based on consensus, and in cases of variation we assigned the higher possible score to obtain a best case scenario. Inter‐rater reliability was assessed with Fleiss’ kappa.^20^, ^21^

Box 3 **Scoring and colour coding system for comparative analysis of the commitment, monitoring, and actions of pharmaceutical companies operating in Australia for achieving net zero greenhouse gas emissions**

**Table:** 

Scoring by domain/subdomain	Colour coding system
**A. Standardised accountability (9 points)**	Red: 0–3 Yellow: 4–6 Green: 7–9
A1. Baseline disclosure Baseline disclosures for scope 1, 2, 3 emissions: 1 point each	
A2. Progress disclosure Reductions in scope 1, 2, 3 emissions: 1 point each (increased or non‐disclosure of emissions: 0 points).	
A3. Standardised disclosure CDP grade A: 3 points; CDP grade B or C: 2 points; CDP grade D or E: 1 point; CDP grade F or none disclosed: 0 points.	
**B. Validated ambition (11 points)**	Red: 0–3 Yellow: 4–7 Green: 8–11
B1. Science‐based targets SBTi‐validated net zero emissions target: 3 points; awaiting validation or committed to target:* 2 points; non‐SBTi target: 1 point; no net zero emissions target: 0 point. SBTi‐validated interim target: 1 point. Specific scope 1, 2, 3 targets: 1 point each.	
B2. Mitigation hierarchy Evidence of specific or measurable abatement plans: 1 point; no evidence or non‐specific and non‐measurable abatement plans: 0 point.	
B3. Specific commitments Each commitment to decarbonise a specific area of future business activity including specific timelines: 1 point (maximum of 3 points).	
**C. Quantifiable actions (12 points)**	Red: 0–4 Yellow: 5–8 Green: 9–12
C1. Scope 1 and 2 action Changes to operations or infrastructure to reduce scope 1 and 2 emissions: 1 point each.	
C2. Scope 3 action Change to operations or infrastructure to reduce scope 3emissions: 1 point.	
C3. Ancillary action Ancillary action to reduce emissions (governance, engagement, finance, innovation, offsetting): 1 point each	

CDP = Carbon Disclosure Project grading. SBTi = Science Based Target Initiative.

* Have made a public commitment to setting SBTi targets within 24 months.

### Ethics approval

The Austin Health Discovery and Innovation Unit provided written exemption from formal ethics approval for this study.

## Results

During the 2022–23 financial year, the ten pharmaceutical companies operating in Australia included in our analysis received $7.60 billion from the PBS, or 44.7% of total PBS expenditure on medications (Box 4). We assessed the full text of 221 of 992 records identified by our searches, and included 158 that met our inclusion criteria in our analysis (Box 5).

Box 4 Ten largest pharmaceutical companies operating in Australia, as defined by total amount received for Pharmaceutical Benefits Scheme (PBS)‐subsidised medications*

**Table:** 

			Amount received from PBS, 2022–23	
Company	Parent company (headquarters)	Trading status (stock exchange, symbol)	Absolute	Proportion of PBS expenditure^†^
Janssen‐Cilag	Johnson & Johnson Services (USA)	Public (NYSE: JNJ)	$1 151 263 284	6.77%
Novartis Pharmaceuticals Australia	Novartis (Switzerland)	Public (SIX: NOVN; NYSE: NVS)	$1 091 720 730	6.42%
Abbvie	AbbVie (USA)	Public (NYSE: ABBV)	$782 766 506	4.60%
Arrotex Pharmaceuticals	Arrotex Holdings (Australia)	Private	$782 437 914	4.60%
Bayer Australia	Bayer (Germany)	Public (FRA: BAYN)	$744 590 992	4.38%
Merck Sharp & Dohme (Australia)	Merck & Co (USA)	Public (NYSE: MRK)	$674 891 157	3.97%
AstraZeneca	AstraZeneca (UK)	Public (LSE: AZN; NASDAQ: AZN)	$670 125 411	3.94%
Vertex Pharmaceuticals (Australia)	Vertex Pharmaceuticals (USA)	Public (NASDAQ: VRTX)	$626 491 783	3.69%
Alphapharm	Viatris (USA)	Public (NASDAQ: VTRS)	$587 548 945	3.46%
Roche Products	Roche Holding (Switzerland)	Public (SIX: ROG)	$486 942 238	2.86%
*Total for ten companies*			*$7 598 778 960*	*44.7%*

FRA = Frankfurt Stock Exchange; LSE = London Stock Exchange; NASDAQ = National Association of Securities Dealers Automated Quotations; NYSE = New York Stock Exchange; SIX = Swiss Infrastructure Exchange; UK = United Kingdom; USA = United States of America.

* As reported in the PBS expenditure and prescriptions report for 1 July 2020 – 30 June 2021,^17^ and updated using the 2022–23 report.^2^ Sandoz removed from tenth position because of spin‐off from Novartis in October 2023; Roche was included in its place.

† Total government expenses for the supply of medications, 2022–23: $17 billion.

Box 5 **Identification and assessment of pharmaceutical company documents published 12 December 2015 – 31 December 2023 for inclusion in our assessment of commitment, monitoring, and actions for achieving net zero greenhouse gas emissions**

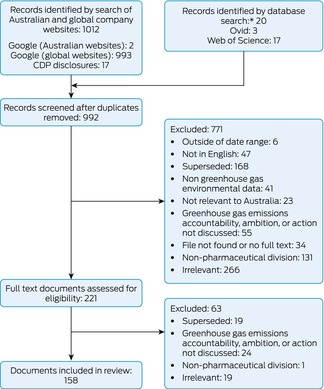

CDP = Carbon Disclosure Project.

* Database search for period 12 December 2015 to 31 August 2022 only.

Five companies scored highly across all three domains (24 of 32 points or better). The highest overall score was for the seventh largest PBS beneficiary, AstraZeneca (30 of 32 points); that is, its performance with respect to decarbonisation targets, monitoring, and actions to reduce emissions was the best of the ten companies. Arrotex Pharmaceuticals scored 0 points because no publicly available data for the study period were identified (Box 6). Inter‐rater reliability for scoring was substantial (κ = 0.78; 95% confidence interval, 0.65–0.90).

Box 6 Assessment the ten largest pharmaceutical companies operating in Australia for commitment to, monitoring of, and actions for achieving net zero greenhouse gas emissions, 2015–23*

**Table:** 

Parent company	Standardised accountability	Validated ambition	Quantifiable actions	Total score
*Maximum score possible*	*9*	*11*	*12*	*32*
AstraZeneca	8	11	11	30
Novartis	8	10	10	28
Johnson & Johnson Services	8	9	11	28
Bayer	8	10	9	27
Merck & Co.	7	10	9	26
AbbVie	7	6	9	22
Roche Holding	4	9	7	20
Viatris	7	4	8	19
Vertex Pharmaceuticals	7	0	5	12
Arrotex Pharmaceuticals	0	0	0	0

* See Box 3 for scoring criteria. Full ratings for each domain are included in the Supporting Information, table 1.

Nine companies (exception: Arrotex Pharmaceuticals) conducted baseline measurements of scope 1 to 3 emissions during 2015–21, and the same nine reported emissions reductions over time. Commitments to SBTi net zero emission targets for scope 1 to 3 emissions between 2040 and 2050 were approved for four companies, and eight companies had set interim targets, with differing levels and timeframes for change (Supporting Information, tables 1 and 2). The leading companies achieved the largest scope 1 and 2 emissions reductions from baseline (AstraZeneca, 68% from 2015 to 2023; Novartis, 63% from 2016 to 2023; Johnson & Johnson, 41% from 2016 to 2022). Six companies conducted and reported scope 3 baseline measurements; four reported that they were higher than at baseline or for earlier reporting periods (AstraZeneca, 18.6% from 2019 to 2023; Johnson & Johnson, 11% from 2016 to 2022; Bayer, 12.5% from 2019 to 2021; Merck, 6% from 2019 to 2022).

Increasing renewable electricity use, improved governance structures, and engagement were the most frequently used quantifiable actions, digitisation and carbon offsetting the least used (Box 7).

Box 7 **Specific quantifiable actions reported in publicly available documents by ten largest pharmaceutical companies operating in Australia, 2015–23: content analysis**

**Table:** 

Scope actions	Companies	Total score (out of 10)
Scope 1 and 2		
Renewable electricity	AstraZeneca, Novartis, Johnson & Johnson Services, Bayer, Merck & Co, AbbVie, Roche Holding, Viatris, Vertex Pharmaceuticals	9
Manufacturing process improvements	AstraZeneca, Novartis, Johnson & Johnson Services, Bayer, AbbVie, Roche Holding, Viatris	7
Heating, ventilation, air conditioning improvements	AstraZeneca, AbbVie, Roche Holding, Viatris, Vertex Pharmaceuticals., Merck & Co.	7
Electrification of company fleet	AstraZeneca, Novartis, Johnson & Johnson Services, Bayer, Merck & Co, AbbVie	6
Scope 3		
Supply chain expectations	AstraZeneca, Novartis, Johnson & Johnson Services, Bayer, Merck & Co, AbbVie, Roche Holding, Viatris	8
Business‐related travel reduction	AstraZeneca, Novartis, Johnson & Johnson Services, Bayer, Merck & Co, Viatris, Vertex Pharmaceuticals	7
Waste disposal improvements	AstraZeneca, Johnson & Johnson Services, Merck & Co, AbbVie, Roche Holding, Viatris, Vertex Pharmaceuticals	7
Digitisation	Novartis	1
Ancillary emission reduction actions		
Governance	AstraZeneca, Novartis, Johnson & Johnson Services, Bayer, Merck & Co., AbbVie., Roche Holding, Viatris., Vertex Pharmaceuticals	9
Engagement	AstraZeneca, Novartis, Johnson & Johnson Services, Bayer, Merck & Co, AbbVie, Roche Holding, Viatris, Vertex Pharmaceuticals	9
Financing	AstraZeneca, Novartis, Johnson & Johnson Services, Bayer, Merck & Co, AbbVie	6
Offsetting	AstraZeneca, Novartis, Johnson & Johnson Services, Bayer	4

## Discussion

We undertook a qualitative assessment of the commitments, accountability, and quantifiable actions for achieving net zero greenhouse gas emissions by the ten largest pharmaceutical companies operating in Australia. Based on our findings, three groups could be defined. The first — companies leading emissions reduction efforts with SBTi‐approved near term targets, consistent emissions monitoring, well defined commitments, and quantified evidence of action — includes AstraZeneca, Novartis, Johnson & Johnson, Bayer, and Merck & Co.; the second group — companies that had made commitments to SBTi‐approved targets but their disclosure records are limited — includes AbbVie and Roche; and the third group — without public commitments to achieving net zero emissions, minimal or no SBTi‐approved targets, and minimal disclosure or monitoring of emissions — includes Viatris, Vertex, and Arrotex.

Scope 1 and 2 emissions reductions were larger for companies in the first group than in their baseline reporting years (Supporting Information, table 1). These reductions are similar to those reported by a recent study that examined the emissions targets of the twenty largest global pharmaceutical companies: six had reduced their scope 1 emissions by more than 20% from their respective baseline year of reporting, and eight companies had reduced scope 2 emissions by 30% or more.^15^ This indicates that reaching company targets (especially for scope 1 and 2 emissions) is possible, but achieving the published net zero emissions reduction targets across scopes 1 to 3 within the next two to eleven years, as planned, could require more rapid reductions.

AstraZeneca achieved the highest score in our assessment (30 of 32 points), meeting all criteria, including long term targets, SBTi approval for their 2045 net zero emissions goal, and defined baseline years for all three emission scopes. AstraZeneca has clear commitments with defined timeframes, including transitioning to 100% renewable electricity by 2025 and expecting 95% of suppliers to have science‐based targets according to Paris Agreement‐aligned pathways. They used multiple methods to reduce emissions, with progress toward renewable electricity for power and heating, electrification of fleet vehicles, changing manufacturing processes and waste disposal to replace natural gas, establishing supply chain expectations with its suppliers regarding greenhouse gas emission targets, less business‐related travel, heating and cooling efficiency measures, employee engagement programs, and governance structures that oversee progress toward emission reduction targets.

The two companies with the second highest scores, Novartis and Johnson & Johnson, are each larger than AstraZeneca, respectively receiving 6.42% and 6.77% of total PBS expenditure for medications during 2022–23 (AstraZeneca: 3.94%). Novartis, but not Johnson & Johnson, has committed to an SBTi‐approved net zero emissions target, and has more recently reported an absolute reduction in scope 1 and 2 emissions from baseline.

As larger companies generally have larger emissions profiles, they need to achieve larger absolute reductions to achieve the same proportional emissions reduction targets over time. Smaller steps by larger companies toward net zero emissions may involve greater absolute reductions. Less ambitious commitments by other companies, particularly to science‐based net zero emissions targets, was associated with incomplete monitoring of emissions, less detail about decarbonisation strategies, less specific timeframes for ambitions, and unclear rationales for stated or intended actions.

Arrotex Pharmaceuticals, the only private company among the ten largest pharmaceutical companies, did not meet any of our criteria; no CDP grades were submitted, no net zero or emissions reduction targets, abatement plans, or decarbonisation strategies were available, and no action for any emission scope had been reported. Arrotex received a larger proportion of PBS medications expenditure than all but three of the companies in our analysis; it is the largest producer of generic medicines in Australia, supplying about one‐third of PBS‐subsidised prescription medications (about 70 million units) each year.^22^ No data are publicly available, but Arrotex (an Australian company) could reduce scope 3 emissions by proposed moves to manufacturing medications in Australia.^23^ Given medication shortages in Australia and reliance on imported medications, local manufacturing should be supported, but it should be environmentally sustainable.

Private or unlisted companies are not subject to public shareholder scrutiny. Shareholders increasingly support reducing environmental impact as part of corporate social responsibility, including improved transparency and accountability.^24^ For example, the 2021 AbbVie annual report noted shareholder concern about misalignment of its stated position on climate change and its donations to political parties.^25^ Standardised reporting measures, such as the CDP grading available to all companies, are important not just as impact measures but also for increasing disclosure transparency for shareholders and the public.^26^ Eight of the ten largest pharmaceutical companies operating in Australia (exceptions Roche, Arrotex) had reported data for assessment and scoring according to the CDP initiative.

Most actions undertaken by the ten companies aimed to reduce scope 1 and 2 emissions, most frequently improved manufacturing processes, heating and cooling efficiency, and electrification of the company fleet, as well as transitioning to electricity produced from renewable sources, which was reported by all nine public companies. However, scope 3 emissions across the entire value chain of a company, from upstream suppliers to end‐of‐life treatment of products, typically comprise the largest proportion of emissions.^15^ Only six companies had conducted and reported scope 3 baseline measurements; similarly, in the recent Oxford team analysis, eleven of the top twenty global pharmaceutical companies were comprehensively reporting scope 3 emissions.^15^ In our study, four companies reported that scope 3 emissions were higher than at baseline or for earlier reporting periods. Explanations for the increases may include business growth^27^ and adjustments in the assessment methodology,^28^ but the changes could also indicate that clear and universal standards for defining and measuring emissions across all industries are needed.

Difficulties for pharmaceutical companies in measuring and reducing scope 3 emissions have been acknowledged.^29^ Several international frameworks have included science‐aligned solutions to reduce them,^30^ including engaging suppliers with smaller emissions profiles, adopting circular economy principles, embedding sustainability key performance indicators into procurement policies, and pricing carbon to provide a financial incentive for low carbon business models. Our study focused on quantitative analysis of actions being undertaken by pharmaceutical companies, and further work is required to qualitatively assess the extent to which the ten largest companies are applying environmental best practice. Reducing scope 3 emissions will ultimately require meaningful engagement with both suppliers and customers.

There is no regulatory framework for climate reporting by pharmaceutical companies in Australia, but we noted a change in emissions reporting practices during the period of our analysis, particularly during 2021–2023. It probably reflects changes in external motivators, such as corporate branding and stronger international legislation regarding mandatory reporting, as well as internal motivators, such as recognition of cost savings, improved research and development processes, and leadership that promotes sustainability and strategic management.^15^ Mandatory disclosure of emissions has been introduced in Europe, requiring large public companies to report on their environmental sustainability.^12^ At the industry level, several international pharmaceutical trade associations have co‐released a joint statement on developing more sustainable supply chains.^31^ The proposed amendment to the Australian *Corporations Act 2001* would require corporations to publish climate disclosures from 2025,^11^ including reporting on reductions of scope 1 to 3 emissions, governance, strategy, risk and transition planning, aligning Australian regulations with those of other countries, and increasing transparency for shareholders and the public, without limiting voluntary disclosure by corporations.

In England, the *Delivering a “net zero” National Health Service* report exemplifies how health sector‐wide leadership in decarbonisation has advanced corporate emissions reduction.^32^ The NHS plan has been referenced as justification for improved environmental metrics by AstraZeneca^33^ and Johnson & Johnson,^34^ two companies that scored well in our analysis. Standardised reporting, sector leadership, and transparent disclosure requirements play influential roles in emissions reduction ambitions, and provide pathways for pharmaceutical companies operating in Australia to improve their net zero emissions plans.

To support such change, improved health sector leadership and an Australian regulatory framework are needed. The newly established National Health, Sustainability and Climate Unit and the National Health and Climate Strategy^35^ are important for developing further mandatory, independent, objective metrics for decarbonisation. Such a unit and strategy could increase Australian pharmaceutical sector sustainability as companies use sustainability reporting to align their practices with regulatory requirements, while continuing to seek a competitive edge.

### Limitations

Our analysis was limited by the availability of published data and voluntary disclosures. Additional actions for environmental sustainability may not have been publicly reported. Conversely, companies may report their environmental efforts in a more favourable light than justified by their performance (“greenwashing”),^36^ a possibility supported by findings that managers adapt their disclosure strategies to different audiences.^26^ For this reason, we used multiple reporting frameworks in our analysis to differentiate between aspiration and progress. We applied a multifaceted scoring system that directly compared targets and actions. We acknowledge that our scoring system might not fully capture the heterogeneity and complexity of the company actions disclosed, nor their efficacy in reducing emissions. One framework alone does not provide comprehensive coverage of commitment to achieving net zero emissions, but the companies that scored best in our analysis were also aligned with SBTi and CDP requirements.

Nevertheless, the industry‐non‐specific criteria of our comparative analysis could be useful for appraising other health‐related sectors, including hospitals and medical technology and device companies. This approach could be useful for policymakers and health professionals developing guidelines for and a culture of choosing low carbon options in clinical practice as part of broader strategies to reduce health care sector emissions, particularly as health systems, networks, and hospitals in Australia begin to publicise their commitments to net zero greenhouse gas emissions.^37^

### Conclusion

We found that the ten largest pharmaceutical companies operating in Australia are moving towards net zero greenhouse gas emissions at different rates. We identified gaps in standardised reporting processes that should be closed to achieve both corporate accountability in achieving verifiable emissions reductions and bringing the Australian health system in line with those of other Western high income nations. Our findings indicate that more research and government support for standardised reporting frameworks for corporate decarbonisation strategies is required. Finally, our evaluation can assist clinicians make informed decisions about low carbon suppliers of medicines, and indicates the need for industry change in transitioning to a sustainable Australian health care sector.

## Competing interests

All authors are members of Doctors for the Environment Australia.

## Data sharing

We confirm that the data supporting the findings of this study were derived from public domain sources and are available in the article and its supplementary materials.

## Supporting information


Supplementary results
