# Supplementary material for: Accountability, ambition, and quantifiable action in the carbon emission reduction plans of the ten largest pharmaceutical companies in Australia: a cross‐sectional analysis
Source: Med J Aust. 2025 Mar 4;222(6):305–12. doi: 10.5694/mja2.52621 (PMC11972594; doi:10.5694/mja2.52621)
Supplement: Supplementary file 1 — Supplementary results [file MJA2-222-305-s001.pdf]

Supporting Information

Supplementary results

This appendix was part of the submitted manuscript and has been peer reviewed.
It is posted as supplied by the authors.

Appendix to: Burch H, Brown G, Adler O, et al. Accountability, ambition, and quantifiable action in the carbon emission reduction plans of the ten largest pharmaceutical companies in Australia: a cross-sectional analysis. *Med J Aust* 2025; doi: 10.5694/mja2.52621.

Table 1. Commitment, monitoring, and actions to achieve net zero greenhouse gas emissions by ten leading pharmaceutical companies (2022–23 Pharmaceutical Benefits Schedule payments), 2015–23

**Parent Company**	**Accountability**			**Ambition**			**Action – refer to table below.**		
	**Baseline disclosure**	**Progress disclosed (*From baseline to year stated in brackets unless otherwise identified*)**	**Standardised disclosure**	**Science-based targets**	**Mitigation hierarchy**	**Specific commitments**	**Scope 1 and 2**	**Scope 3**	**Ancillary actions**
AstraZeneca	Baseline:<br>S1-2: 2015<br><br>S3: 2019	S1, S2: ↓ 67.6% (2023)<br>Δ420ktCO2e<br><br>S3: ↑ 18.6% (2023)<br>Δ1056ktCO2e	CDP: 2023 (A)	SBTi approved targets:<br><br>Net zero by 2045<br><br>S1-2: 98% by 2026<br><br>S3: 50% by 2030; 90% by 2045	Emissions sources to be eliminated from 2030 with residual emissions compensated through nature-based solutions and bioenergy with carbon capture and storage	100% electric vehicle fleet by 2025 – EV100<br><br>100% renewable electricity and heat by 2025 – RE100<br><br>Energy consumption reduction by 10% and energy productivity to double by 2025 – EP100<br><br>Near-zero global warming potential propellant metred dose inhalers by 2030<br><br>95% of suppliers to have 1.5C Science Based Targets by 2025<br><br>46% GHGE reduction in purchased goods and services by 2030<br><br>46% GHGE reduction in upstream transportation and business travel 2030<br><br>80% reduction in fuel and energy related GHGE<br><br>Footprint from materials used by patients to decrease by 95% 2030.<br><br>Over 50 million trees to be planted by 2025 through AstraZeneca Forest Global Initiative	All AstraZeneca labs certified by My Green Lab<br><br>30 chillers at Swedish manufacturing site replaced with low GWP models<br><br>35% of fleet are electric battery vehicles, 75% green fleet (hybrid +EV)<br><br>95% of all electricity used came from renewables<br><br>17.5% reduction in total energy consumption since 2015<br><br>$19.4M invested in solar installations across 9 facilities to provide 4.1% of total energy needs.<br>Renewable power agreement in China with potential savings of 120,000 tons CO2<br><br>PPA with wind farm in Sweden to provide 200GWh<br><br>New research and development site in Cambridge built with green heating, ventilation and air-conditioning (HVAC) engineering	Respiratory products switched to paper-based trays<br><br>97.5% paper-based product packaging from suppliers from sustainable sources<br><br>13.2% reduction in waste from 2015<br><br>Supply chain expected to set SBTs, annually report through EcoVadis and CDP, and minimise GHGE<br><br>Business air travel emissions reduced by 52%<br>Transition of freight from air to land and sea.	Board and executive oversight of climate-related strategy with financial incentives. Executive led Ambition Zero Carbon governance group including CEO, CFO and chief sustainability officer.<br><br>AstraZeneca forest program. $400M invested. >19.9m trees planted tthrough forestry projects in Australia, Indonesia, the UK, Ghana and US<br><br>175M invested in natural resource efficiency projects since 2015.<br><br>$33.7M invested in on-site renewable energy and energy efficiency.<br><br>Pharmaceutical Supply Chain Initiative (PSCI), Sustainable Markets Initiative (SMI) Health Systems Taskforce, Race to Zero, EV100, RE100, EP100, Energize program (Supply Chain Renewables Initiative)

Parent Company	Accountability			Ambition			Action – refer to table below.		
	**Baseline disclosure**	**Progress disclosed (*From baseline to year stated in brackets unless otherwise identified*)**	**Standardised disclosure**	**Science-based targets**	**Mitigation hierarchy**	**Specific commitments**	**Scope 1 and 2**	**Scope 3**	**Ancillary actions**
Novartis AG	Baseline:<br>S1-3 2016	S1, S2: ↓63% (2023)<br>*S1 Δ216ktCO2e*<br>*S2Δ460ktCO2e (market based)*<br><br>S3: No reporting from baseline. 3.4% decrease from 2022 to 2023. 20% increase 2021 to 2022. 10% decrease from 2016 to 2021	CDP: 2023 (A)	SBTi approved targets:<br><br>S1-3: 35% by 2030<br><br>Net zero 2040 – SBTi Committed (Novartis has made a public commitment to align net-zero target with SBTi criteria within 24 months)<br><br>Non-SBTi targets:<br><br>S1-2: 100% carbon neutral in own operations by 2025<br><br>S1-3: 100% carbon neutral across value chain 2030	Achieve 2030 carbon neutrality target by reducing scope 1-3 emissions by at least 35% followed by neutralisation of remaining emissions through carbon avoidance and carbon removal projects	94% EV fleet by 2030<br><br>100% renewable energy by 2025<br><br>Environmental criteria in all supplier contracts by 2025<br><br>All new products meet sustainable design principles by 2030<br><br>Third party code – all products and services precured by Novartis to be carbon neutral by 2030.<br><br>Plastic neutral by 2030<br><br>Reduce the amount of waste sent for disposal by 50% by 2025	24 laboratories certified by My Green Lab<br><br>Manufacturing efficiency improved by continuous manufacturing, biocatalysts, high-intensity perfusion batches<br><br>Thermal emissions improvements: conversion to electric boilers (renewable electricity operated), solar thermal installation, biomass-based steam generation.<br><br>EV fleet – as of 2022, 10.5% of vehicles battery electric.<br><br>100% renewable electricity in North America, and Europe through VPPAs<br><br>92% purchased electricity from renewable sources	Replaced product information leaflets with electronic forms<br><br>Supply chain expected to monitor GHGE, set 2025 and 2030 targets disclose through CDP or EcoVadis and PSCI (24, 32)<br>57% of supplier emissions covered by contracts that include environmental sustainability criteria<br><br>Environmental Sustainability Supplier Playbook (comprehensive guidance to suppliers on transitioning to sustainable business models)<br><br>Green Logistics program transition from air to sea shipments<br><br>Transitioned to virtual meetings to reduce business travel	Board and executive oversight of climate-related strategy<br><br>Financing: Internal carbon pricing of 100 USD/tCO2e into strategic decision planning<br><br>Capital expenditure (2023) of 25.5 million on environmental projects.<br><br>Forestry projects in Argentina, Mali, China and Colombia<br><br>SBTi Business Ambition for 1.5°C, Race to Zero, RE100, EV100, Ellen MacArthur Foundation and Sustainability 30 (S30), Energize, PSCI, Task Force on Climate Related Financial Discourses (TFCFD)
Johnson & Johnson Services Inc.	Baseline:<br>S1-3 2016	S1, S2: 41% reduction (2022)<br>Δ483ktCO2e<br><br>S3: 11% increase (2022)<br><br>40% reduction in carbon footprint (2022)	CDP 2023 (A)	SBTi approved targets:<br><br>S1-2: 60% by 2030<br><br>S3: 20% by 2030.<br><br>Non-SBTi targets:<br><br>Net zero ambition by 2045<br><br>Carbon neutrality by 2030	Investment in carbon compensation and removal partnerships<br><br>Remaining carbon emissions to be balanced by sequestration	Hydrochlorofluorocarbon (HCFC) use to be eliminated by 2025<br><br>100% renewable energy by 2025<br><br>Supplier sustainability programme for all suppliers<br><br>Direct PPA for 100% renewable energy for operations in Brazil, United States, Europe, Canada.<br><br>Third party operations to become carbon neutral by 2030	Eight sites in 2022 received Leadership in Energy & Environmental Design (LEED) certifications<br><br>6 sites certified carbon neutral<br><br>My Green Lab certification for two facilities.<br><br>67% of electricity produced or procured from renewable energy sources.<br><br>Energy intensity (TJ/$1bn) reduced by 30%<br><br>Direct PPAs in effect in Europe, USA, Mexico, India, and Brazil (2022)<br><br>Electrification of vehicle fleet.	Conversion of air deliveries to road in Belgium, Denmark, France, Spain<br><br>Rolling Highway between italy and Belgium – Rail transport for fully loaded trucks.<br><br>2022 - 85% of suppliers completed CDP supply chain disclosures<br><br>Supply chain expected to complete EcoVadis assessment and annual CDP Supply Chain disclosures<br><br>Surgical equipment recycling to 8 European countries (Components of 25,000 single use products recucled 2021-22)<br><br>Digital Health in a Circular Economy – Aim to decrease digital health device waste.	Board and executive oversight of climate-related strategy<br><br>$40m per year for energy projects (CO_2 Capital Relief Program)<br><br>Clean Energy Buyers Alliance, SBTi Business Ambition for 1.5°C, Energize, International Leadership Group for a Net Zero National Health Service, We Mean Business Coalition, PSCI<br><br>Forest conservation fund (Peru) – protect 10,000 hectares of Amazon rainforest. World wildlife fund – conserve 38,000 hectares of rainforest in Indonesia.

Parent Company	Accountability			Ambition			Action – refer to table below.		
	**Baseline disclosure**	**Progress disclosed (*From baseline to year stated in brackets unless otherwise identified*)**	**Standardised disclosure**	**Science-based targets**	**Mitigation hierarchy**	**Specific commitments**	**Scope 1 and 2**	**Scope 3**	**Ancillary actions**
Bayer AG	Baseline:<br>S1-3 2019	S1, S2: 19.5% reduction (2022) Δ470ktCO2e Available figures from 2020 to 2022 only. Total ktCo2 change may not indicate 19.5% decrease.)<br><br>S3: 12.% increase from 2021	CDP: 2022 (A)	SBTi approved targets:<br><br>S1-2: 42% by 2029<br><br>S3 (selective): 12% by 2029<br><br>Net zero by 2050 – SBTi committed (Bayer has made a public commitment to align net-zero target with SBTi criteria within 24 months)	Emissions to be first avoided then minimised and offset by carbon credits focused on nature-based solutions<br><br>Carbon credits to espouse principles of transparency, additionality, permanence, measurability, quality / standards, innovation, impact and co-benefits	Carbon neutrality at all own sites by 2030<br><br>Global vehicle fleet to be transitioned to EV by 2030<br><br>100% renewable energy by 2030<br><br>Net zero deforestation in supply chain<br><br>500M euro investment in energy efficiency and climate-friendly measures until 2030<br><br>All customer health product packaging to be 100% recyclable or reusable.	Geothermal energy, emissions-free steam production implemented at various sites<br><br>32.6% of electricity procured from renewable sources<br><br>18% vehicle fleet electric or hybrid.	Supply chain expected to undergo decarbonisation in line with Paris Agreement monitoring GHGE, setting SBTs, adhering to circular economy principles and disclosing through CDP<br><br>Preference of rail and waterway logistics over air transportation	Board and executive oversight with independent advisory sustainability council and incentives.<br><br>700M euro for GHGE reduction projects. Internal Carbon Price of 100€/t<br><br>SBTi Business Ambition for 1.5°C, TCFD, EV100, CDP Supply chain initiative, Climate Action 100+, World Business Council for Sustainable Development (WBCSD), EcoTransIT World Initiative.<br><br>Forestry projects in 15 countries. Tree planting projects in Finland Argentina, Brazil, Mexico, Italy Guatemala.<br><br>Lowering Emissions by Accelerating Forest finance (LEAF) Coalition (mobilized more than US$1.5 billion since 2021 for protection of rainforests)
Merck & Co.	Baseline<br>S1-3: 2019	S1-2: 9% reduction (2022)<br>Δ92ktCO2e<br><br>S3: 6% increase (2022)<br>Δ407ktCO2e	CDP 2023 (B)	SBTi approved targets:<br><br>S1-2: 46% by 2030<br><br>S3: 30% by 2030<br><br>Net zero by 2050 – SBTi committed (Merck and Co have made a public commitment to align net-zero target with SBTi criteria within 24 months)<br><br>Non- SBTi targets:<br><br>S1-2: Carbon neutrality across operations by 2025	Carbon neutral in own operations (S1-2) with remaining emissions to be offset with nature-based or technology-based carbon offsets.	100% renewable electricity by 2025<br><br>By 2025, 90% of suppliers will set own GHGE reduction targets<br><br>By 2025, max 20% global operational waste to be sent to landfill or incinerators<br><br>Road map to transition to full battery electric fleet<br><br>All laboratories and Offices to meet GOLD LEED certification	45% purchased electricity from renewables.<br><br>3 new PPAs purchasing wind and solar form Texas and Spain.<br><br>Solar arrays at Australia and Singapore site<br><br>7 sites with LEED silver/gold certification.<br><br>San Francisco site LEED Zero certification.<br><br>Global energy score card issued monthly for sites assessing energy efficiency and performance<br><br>3.7M square foot of 'Green' floor space completed or being constructed.<br><br>Hybrid fleets - 12% Europe, Middle East and Africa; 56% in Japan, China and Asia Pacific; 1% in Latin America; 2% North America	80% waste recycled/reused.<br><br>60% of waste diverted from landfill<br><br>48% of sites zero landfill.<br><br>Simplified life-cycle assessment (LCA) to evaluate environmental impact of packaging.<br><br>Rail travel option for all business- travel bookings Improved shipment consolidation and shift away from air freight<br><br>GHGE data collected from 90% of suppliers. 52% suppliers have GHG emissions targets	Environmental Health and Safety Council with senior level executives. Environmental Sustainability Centre of Excellence (CoE) + Global Energy & Sustainability CoE oversees climate goals/targets/actions.<br><br>PSCI, We Mean Business Coalition, SBTi Business Ambition for 1.5°C, ENERGISE program, Pharmaceutical Environment Group (PEG)<br><br>$12M/year invested in low carbon technology through Sustainability Capital Fund.

Parent Company	Accountability			Ambition			Action – refer to table below.		
	**Baseline disclosure**	**Progress disclosed (*From baseline to year stated in brackets unless otherwise identified*)**	**Standardised disclosure**	**Science-based targets**	**Mitigation hierarchy**	**Specific commitments**	**Scope 1 and 2**	**Scope 3**	**Ancillary actions**
AbbVie Inc.	Baseline<br>S1-3: 2021	S1-2**:** 15.6% reduction (2022)<br><br>S1: Δ25.3ktCO2e<br>S2: Δ72.2 ktCO2e (Market based)<br><br>No S3 data	CDP: 2023 (B)	SBTi approved targets:<br><br>S1-2: 42% 2030<br><br>*No net zero or S3 target*	None	41% reduction of scope 1 emission from vehicle fleet by 2030<br><br>50% of electricity to be sourced by renewables by 2025, 100% by 2030<br><br>79% of suppliers to have science-based targets by 2027<br><br>20% reduction in waste by 2025<br><br>Global facility design guidelines to be used for new, sustainably designed buildings	New LEED Platinum certified R&D facility in San Francisco<br><br>Co-generation system in Puerto Rican site and all steam boilers in Italian site replaced<br><br>Improvements in heating ventilation and air-conditioning<br><br>42.7% electricity produced from renewable sources, 100% at European manufacturing sites<br><br>9.6% reduction in energy consumption.<br><br>PV solar system at Belgium site providing 1/3 yearly energy<br><br>95% of new vehicles introduced in UK fleet were EV or hybrid; >30% of those introduced to western Europe fleet. 3000 electric vehicles total.	Packaging changes reduced GHGE from waste disposal by 98%<br><br>92% of waste diverted from landfill<br><br>89% of suppliers surveyed have set goals and strategies to reduce energy use and GHGE<br><br>78% of suppliers have a formal environmental/ sustainability plan through supplier engagement program<br><br>23% of suppliers established science-based reduction targets	Board and executive oversight of climate-related strategy with financial incentives<br><br>SPARK Innovation Accelerator to foster employee-driven ideas to reduce energy usage<br><br>SBTi Business Ambition for 1.5°C, PSCI, TCFD, S&P Global Trucost<br><br>Corporate energy Capital fund - 28 energy reduction and energy resilience projects funded – $5.8M 2022.<br><br>Corporate Decarbonization Capital fund - $4M 2022. Funded 21 projects.

Parent Company	Accountability			Ambition			Action – refer to table below.		
	**Baseline disclosure**	**Progress disclosed (*From baseline to year stated in brackets unless otherwise identified*)**	**Standardised disclosure**	**Science-based targets**	**Mitigation hierarchy**	**Specific commitments**	**Scope 1 and 2**	**Scope 3**	**Ancillary actions**
Roche Holding AG	Baseline:<br>S1-2: Unclear<br><br>S3 2019	S1-2: 29% decrease since 2019, 73% reduction since 2004.<br><br>S1-2: 0.9% tCO_2/employee decrease<br><br>S3: 24% decrease from 2019 to end 2021. 8% increase across 2022.	CDP: 2023 (F) – No response	Net zero 2050 – SBTi committed (Roche Holding AG has made a public commitment to align target with SBTi criteria for net zero emissions within 24 months)<br><br>Non – SBTi targets<br><br>S1-2: zero emissions by 2050<br><br>S1-2 40% tCO_2/employee by 2025 (2020 baseline)<br><br>S1-2 75% by 2029 (2019 baseline)<br><br>S3: 18% tCO_2/employee by 2025<br><br>S1-2 energy consumption - 10% decrease by 2025<br><br>Reduce total environmental impact by 50% from 2019-2029	Operational emissions to be avoided or reduced or substituted instead of compensating with offsets.	100% sustainable electricity by 2025<br><br>All emissions form cooling and refrigeration to be eliminated by 2030<br><br>15% reduction in Scope 3 GHGE intensity per employee by 2025<br><br>Sites to develop a roadmap towards Net zero S1-2<br><br>18% reduction of CO_2 from business flights by 2025<br><br>GHG free vehicle fleet by 2030.<br><br>logistics/courier companies to be GHG free by 2040.<br><br>Minimum supplier targets including assessing and disclosing S1-3 emissions (2025) and committing to SBTs (2025)<br><br>Eliminated all halogenated hydrocarbons from Roche own and leased facilities by 2030	81% of electricity procured from renewable sources<br><br>Energy consumption improved at Basel and Kaiseraugst sites by green HVAC design<br><br>Increased percentages of renewable energy used at sites in US (52 → 100%) and Japan (36→87%)<br><br>Sustainable building designs (basal and San Francisco)<br><br>Natural refrigerant heat pumps, solar façade at Oceanside site.<br><br>Geothermal technology at Branchburg site and Penzburg site.<br><br>Woodfired heating plant using forestry waste for steam production at Swiss site	Joint minimum climate and sustainability targets (SMI)<br><br>Procurement organisation and Product Stewardship program assisted key suppliers in reducing their footprint.<br><br>>80% of general waste to be recycled 2025.<br><br>10% Reduction in general waste 2025	Board and executive oversight of climate-related strategy with financial incentives.<br>Individual sites define local goals and action plans<br><br>SBTi Business Ambition for 1.5°C campaign, Biopharma Sustainability Roundtable, Sustainable market Initiatives, ENERGISE renewable electricity program, PSCI, TCFD<br><br>Partnership with E.ON group to source 100% renewable electricity
Viatris Inc.	Baseline:<br>S1-3: 2020	S1: 4% decrease (2022) Δ13.5ktCO_2e<br><br>S2: 10% decrease (2022) Δ40.8ktCO_2e<br><br>S3: No reporting	CDP: 2023 (B)	SBTi validated targets:<br><br>S1-2: 42% by 2030<br><br>S3 25% by 2030 (Across purchased goods and services, capital goods, fuel and energy related activities, and upstream transportation and distribution<br><br>*No net-zero target*	None	Renewable energy usage, energy efficiency projects including heat pumps, sustainable refrigerants and preventing leaks, use of alternative fuels to be increased *(No specific timeline or target)*<br><br>Support of suppliers to set their own SBTs through PSCI *(No specific timeline or target)*<br><br>Preference of sea transport over flight *(No specific timeline or target)*<br><br>Increase number of zero-waste facilities by 50% from 2020 baseline.	PPA for combined heat and power at Puerto Rican site<br><br>PPA agreement for 34% renewable energy at Hosur site, India<br><br>PPA in Ireland, mainly wind power.<br><br>Rooftop solar panels at API manufacturing site in India<br><br>Ground mounted solar panels in API unit India, 25% of total energy<br><br>Solar system at Carole Park, Australia.<br><br>Transitioned to LED lighting across all sites<br><br>ISO Environmental Management certifications for 24 sites<br><br>Liquid petroleum gas at Hosur facility. 7% decrease in scope 1 emissions (2021-22)	89% of transport by road or ocean. Trucks fully loaded before transport.<br><br>Suppliers expected to monitor and reduce GHGE<br><br>Sludge dryer at API site expected to reduce waste sent to landfill by 70%	Governance:<br>Board and executive oversight of climate-related strategy. Chief sustainability officer and Sustainability manager. Monetary incentives<br><br>PSCI, TCFD

Parent Company	Accountability			Ambition			Action – refer to table below.		
	**Baseline disclosure**	**Progress disclosed (*From baseline to year stated in brackets unless otherwise identified*)**	**Standardised disclosure**	**Science-based targets**	**Mitigation hierarchy**	**Specific commitments**	**Scope 1 and 2**	**Scope 3**	**Ancillary actions**
Vertex Pharmaceuticals Inc.	Baseline<br>S1-2: 2018<br>S3: Nil	S1-2: 21.4% decrease<br>Δ *<br><br>S3: No disclosure	CDP 2023 (A)	No targets beyond 2023<br><br>Target of S1-2 20% reduction met in 2023	None	Leiden Centre to be LEED Platinum certified<br><br>My Green Lab certification applications for Boston and Oxford.	100% renewable electricity for U.K. facilities<br><br>Solar Array power at San Diego facility (20% needs) 83% green-certified square footage in our buildings<br><br>LEED gold certified facilities in San Diego and Boston<br><br>Energy efficiency in buildings including LED lighting at Boston centre.<br><br>Heat recovery and heat pumps at Boston labs	Green Allowance for commuting via public transport.<br><br>60% waste diverted from land fill.	Board of directors to oversee corporate environmental responsibility initiatives. Internal sustainability committee oversees implementation of environmental programs.<br><br>GHGE protocol corporate standard, PSCI, TCFD. EPA's Energy Star program
Arrotex Pharmaceuticals Pty Ltd	Nil baseline	None	CDP: None	No targets	None	None	None	None	None

S1 = Scope 1; S2 = Scope 2; S3 = Scope 3; CDP = Carbon Disclosure project; EV100 = Climate Group EV100; RE100 = Climate Group RE100; EP100 = Climate Group EP100; SBTi = Science Based Targets Initiative; GHGE = Green House Gas Emissions; GWP = Global Warming Potential; PPA = Power Purchase Agreement; CEO = Chief Executive Officer; CFO = Chief Financial Officer; PSCI = Pharmaceutical Supply Chain Initiative; SMI = Sustainable Markets Initiative; EV = Electric Vehicle; TFCFD = Task Force on Climate Related Financial Discourses; HCFC = Hydrochlorofluorocarbon; LEED = Leadership in Energy & Environmental Design; WBCSD = World Business Council for Sustainable Development; PEG = Pharmaceutical Environment Group; HVAC = Heating, ventilation, and air conditioning; API = Active Pharmaceutical Ingredients; LED = Light Emitting Diode; ISO = International Organisation of Standardisation; EPA = Environmental Protection Agency (USA).

Table 2. Summarised findings from Science Based Target Initiative dashboard, 2022-23 (9)

Parent Company	*Short Term SBTi Validated Targets (Temperature alignment)*	*Long Term SBTi Validated Targets*	*Net – Zero SBTi Validated Target*
**Johnson & Johnson Services**	• S1-2: 60% by 2030 (1.5°C)<br>• S3: 20% by 2030 (1.5°C)	X	Commitment removed
**Novartis**	• S1-3: 35% by 2030 (Well-below 2°C)	X	Committed
**AbbVie**	• S1-2: 42% 2030 (1.5°C)	X	X
**Arrotex Pharmaceuticals**	X	X	X
**Bayer**	• S1-2: 42% by 2029 (1.5°C)<br>• S3 (selective): 12% by 2029 (1.5°C)	X	Committed
**Merck & Co**	• S1-2: 46% by 2030 (1.5°C)<br>• S3: 30% by 2030 (1.5°C)	X	Committed
**AstraZeneca**	• S1-2: 98% by 2026 (1.5°C)<br>• S3: 50% by 2030 (1.5°C)	S3 90% by 2045	Net Zero by 2045
**Vertex Pharmaceuticals**	X	X	X
**Viatris**	• S1-2: 42% by 2030 (1.5°C)<br>• S3 (selective): 25% 2030 (1.5°C)	X	X
**Roche Holding**	Committed	X	Committed

SBTi = Science Based Targets Initiative; S1 = Scope 1; S2 = Scope 2; S3 = Scope 3.

References

1. Johnson & Johnson. Johnson & Johnson - climate change 2023. London: Carbon Disclosure Project, 2023. https://www.cdp.net/en/formatted_responses/responses?campaign_id=83630982&discloser_id=1033847&locale=en&organization_name=Johnson+%26+Johnson&organization_number=9829&program=Investor&project_year=2023&redirect=https%3A%2F%2Fcdp.credit360.com%2Fsurveys%2F2023%2Fjwbhd7d6%2F271941&survey_id=82591262 (viewed Mar 2024).
2. Novartis. Novartis - climate change 2023. London: Carbon Discloure Project, 2023. https://www.cdp.net/en/formatted_responses/responses?campaign_id=83630982&discloser_id=1028681&locale=en&organization_name=Novartis&organization_number=13524&program=Investor&project_year=2023&redirect=https%3A%2F%2Fcdp.credit360.com%2Fsurveys%2F2023%2Fjwbhd7d6%2F280313&survey_id=82591262 (viewed Mar 2024).
3. AbbVie. AbbVie Inc - climate change 2023. London: Carbon Disclosure Project, 2023. https://www.cdp.net/en/formatted_responses/responses?campaign_id=83630982&discloser_id=1018175&locale=en&organization_name=AbbVie+Inc&organization_number=45103&program=Investor&project_year=2023&redirect=https%3A%2F%2Fcdp.credit360.com%2Fsurveys%2F2023%2Fjwbhd7d6%2F249851&survey_id=82591262 (viewed Mar 2024).
4. Bayer. Bayer AG - climate change 2023. London: Carbon Disclosure Project, 2023. https://www.cdp.net/en/formatted_responses/responses?campaign_id=83630982&discloser_id=1018283&locale=en&organization_name=Bayer+AG&organization_number=1581&program=Investor&project_year=2023&redirect=https%3A%2F%2Fcdp.credit360.com%2Fsurveys%2F2023%2Fjwbhd7d6%2F253444&survey_id=82591262 (viewed Mar 2024).
5. Merck & Co. Merck & Co., Inc - climate change 2023. London: Carbon Disclosure Project, 2023. https://www.cdp.net/en/formatted_responses/responses?campaign_id=83630982&discloser_id=1017964&locale=en&organization_name=Merck+%26+Co.%2C+Inc.&organization_number=11765&program=Investor&project_year=2023&redirect=https%3A%2F%2Fcdp.credit360.com%2Fsurveys%2F2023%2Fjwbhd7d6%2F278115&survey_id=82591262 (viewed March 2024).
6. AstraZeneca. AstraZeneca - climate change 2023. London: Carbon Disclosure Project, 2023. https://www.cdp.net/en/formatted_responses/responses?campaign_id=83630982&discloser_id=1017959&locale=en&organization_name=AstraZeneca&organization_number=1104&program=Investor&project_year=2023&redirect=https%3A%2F%2Fcdp.credit360.com%2Fsurveys%2F2023%2Fjwbhd7d6%2F314018&survey_id=82591262 (viewed Mar 2024).
7. Vertex. Vertex Pharmaceuticals Inc - climate change 2023. London: Carbon Disclosure Project, 2023. https://www.cdp.net/en/formatted_responses/responses?campaign_id=83630982&discloser_id=1040006&locale=en&organization_name=Vertex+Pharmaceuticals+Inc&organization_number=36916&program=Investor&project_year=2023&redirect=https%3A%2F%2Fcdp.credit360.com%2Fsurveys%2F2023%2Fjwbhd7d6%2F294618&survey_id=82591262 (viewed Mar 2024).
8. Viatris. Viatris - climate change 2023. London: Carbon Disclosure Project, 2023 https://www.cdp.net/en/formatted_responses/responses?campaign_id=83630982&discloser_id=1041552&locale=en&organization_name=Viatris&organization_number=12635&program=Investor&project_year=2023&redirect=https%3A%2F%2Fcdp.credit360.com%2Fsurveys%2F2023%2Fjwbhd7d6%2F295421&survey_id=82591262 (viewed Mar 2024).
9. Science Based Targets Initiative. Companies taking action. London: Science Based Targets, 2024. https://sciencebasedtargets.org/companies-taking-action (updated Mar 2024; viewed Mar 2024).
10. Johnson & Johnson. Position on climate action. New Brunswick: Johnson & Johnson, 2023. https://www.jnj.com/about-jnj/policies-and-positions/position-on-climate-action (updated May 2023; viewed Feb 2024).
11. Johnson & Johnson. Climate action at a glance. New Brunswick: Johnson & Johnson, 2023. https://www.jnj.com/environmental-sustainability/climate-action-plan-at-a-glance (viewed Feb 2024).
12. Johnson & Johnson. 2022 health for humanity report. New Brunswick: Johnson & Johnson, 2023. https://healthforhumanityreport.jnj.com/2022/reporting-hub.html (viewed Feburary 2024).
13. Johnson & Johnson. Responsibility standards for suppliers. New Brunswick: Johnson & Johnson, 2022. https://www.jnj.com/suppliers/responsibility-standards-for-suppliers-english (viewed Feb 2024).
14. Johnson & Johnson. Position on stakeholder engagement. New Brunswick: Johnson & Johnson, 2024. https://www.jnj.com/about-jnj/policies-and-positions/our-position-on-stakeholder-engagement (viewed Feb 2024).

15. Novartis AG. Environmental sustainability criteria for suppliers. Basel: Novartis, 2022. https://www.novartis.com/sites/novartis_com/files/novartis-environmental-sustainability-criteria-for-suppliers.pdf (viewed Mar 2024).
16. Novartis AG. Investor update on access and sustainability. Basel: Novartis, 2022. https://www.novartis.com/sites/novartis_com/files/2022-11-novartis-investor-update-access-sustainability.pdf (updated Nov 2022; viewed Mar 2024).
17. Novartis AG. Q4 impact and sustainability update 2023. Basel: Novartis, 2024. https://www.novartis.com/sites/novartiscom/files/novartis-q4-2023-impact-and-sustainability-update.pdf (viewed Mar 2024).
18. Novartis Pharmaceuticals UK. Carbon reduction plan. London: Novartis, 2023. https://www.novartis.com/sites/novartis_uk/files/PPN-0621-Carbon-Reduction-Plan-Novartis_UK-2023-v3.docx.pdf (viewed Mar 2024).
19. Novartis AG. Investor impact and health equity: Novartis 10th annual ESG investor event. Basel: Novartis, 2023. https://www.novartis.com/sites/novartis_com/files/2023-11-novartis-investor-impact-health-equity.pdf (updated Nov 2023; viewed Mar 2024).
20. Novartis AG. Environmental sustainability strategy objectives 2022. Basel: Novartis, 2023. https://www.novartis.com/sites/novartis_com/files/novartis-environmental-sustainability-strategy-objectives.pdf (viewed Mar 2024).
21. Novartis. Task force on climate related financial disclosures report 2023. Basel: Novartis, 2023. https://www.novartis.com/sites/novartis_com/files/task-force-climate-related-financial-disclosures-tcfd-report-2023.pdf (viewed Mar 2024).
22. Novartis AG. Environmental performance indicators 2022. Basel: Novartis, 2023. https://www.novartis.com/sites/novartis_com/files/novartis-environmental-performance-indicators-fy-2022.pdf (updated Sep 2023; viewed Mar 2024).
23. Novartis AG. Third party code. Basel: Novartis, 2023. https://www.novartis.com/sites/novartis_com/files/novartis-third-party-code-v-3.pdf (updated Jan 2023, viewed Mar 2024).
24. Novartis AG. Annual report 2023. Basel: Novartis, 2024. https://www.novartis.com/sites/novartis_com/files/novartis-q2-2023-impact-and-sustainability-update.pdf (viewed Mar 2024).
25. Novartis AG. Novartis in society integrated report 2023. Basel: Novartis, 2024. https://www.novartis.com/sites/novartis_com/files/novartis-integrated-report-2023.pdf (viewed Mar 2024).
26. AbbVie. Environment and safety performance 2022. North Chicargo: AbbVie, 2023. https://www.abbvie.com/content/dam/abbvie-com2/pdfs/about/environment-and-safety-performance-report.pdf (viewed Mar 2024).
27. AbbVie. Our task force on climate-related disclosures. North Chicargo: AbbVie, 2023. https://www.abbvie.com/content/dam/abbvie-com2/pdfs/tcfd-report.pdf (accessed March 2024).
28. AbbVie. Supplier code of conduct. North Chicargo: AbbVie, 2023. https://www.abbvie.com/content/dam/abbvie-com2/pdfs/suppliers/abbvie-supplier-code-of-conduct-brochure.pdf (viewed Mar 2024).
29. AbbVie. Our approach to supply chain management. North Chicargo: AbbVie, 2023. https://www.abbvie.com/content/dam/abbvie-com2/pdfs/suppliers/abbvie-global-supply-chain-management-overview.pdf (viewed Mar 2024).
30. AbbVie. 2022 Annual report on form 10-K and 2023 proxy statement. North Chicargo: AbbVie, 2023. https://investors.abbvie.com/static-files/e3047152-75b3-44b3-95a8-18e4fa991768 (viewed Mar 2024).
31. AbbVie. ESG action report 2022. North Chicargo: AbbVie, 2023. https://www.abbvie.com/content/dam/abbvie-com2/pdfs/abbvie-esg-action-report.pdf (viewed Mar 2024).
32. Bayer AG. Industry association climate engagement review. Leverkusen: Bayer AG, 2022. https://www.bayer.com/sites/default/files/Bayer_Industry%20Association%20Climate%20Engagement_rg_v05_fin.pdf (viewed Mar 2024).
33. Bayer AG. Bayers climate scenarios. Leverkusen: Bayer AG, 2022. https://www.bayer.com/sites/default/files/TCFD%20Bayer%20Website_20230503.pdf (viewed Mar 2024).

34. Bayer AG. A global leader in health & nutrition - investment case. Leverkusen: Bayer AG, 2023. https://www.bayer.com/sites/default/files/2023-08/bayer-ag-investment-case-august-2023-0_1.pdf (viewed Mar 2024).
35. Bayer AG. Industry association climate review 2023. Leverkusen: Bayer AG, 2023. https://www.bayer.com/sites/default/files/Bayer%20Climate%20Review%202023.pdf (viewed Mar 2024).
36. Bayer AG. Our views on climate mitigation in industry and agriculture. Leverkusen: Bayer AG, 2023. https://www.bayer.com/sites/default/files/Bayer_Position_Global_Climate_Policy_FIN.pdf (viewed Mar 2024).
37. Bayer AG. Annual report 2022. Leverkusen: Bayer AG, 2023. https://www.bayer.com/sites/default/files/2023-02/Bayer-Annual-Report-2022.pdf (viewed Mar 2024).
38. Bayer AG. Bayer’s offsetting approach: focusing on emission reduction while building up nature-based solutions. Leverkusen: Bayer AG, 2023. https://www.bayer.com/sites/default/files/2023-02-01-com-offsetting-publicationv4.pdf (updated Feb 2023; viewed Mar 2024).
39. Bayer AG. Bayer sustainability council 2022 report. Leverkusen: Bayer AG, 2023. https://www.bayer.com/sites/default/files/2023-03-31_Sustainability%20Council%20Report%202022_vf.pdf (viewed Mar 2024).
40. Bayer AG. Supplier code of conduct guidance. Leverkusen: Bayer AG, 2023. https://www.bayer.com/sites/default/files/2023-05/English-version.pdf (updated Apr 2023; viewed Mar 2024).
41. Bayer AG. Bayer sustainability report 2022. Leverkusen: Bayer AG, 2023. https://www.bayer.com/sites/default/files/2023-02/Bayer-Sustainability-Report-2022.pdf (viewed Mar 2024).
42. Bayer AG. Corporate governance presentation. Leverkusen: Bayer AG, 2023. https://www.bayer.com/sites/default/files/corporate-governance-presentation-2023.pdf (updated Apr 2023; viewed Mar 2024).
43. Bayer AG. Investor conference call - ESG update 2022. Leverkusen: Bayer AG, 2023. https://www.bayer.com/sites/default/files/2023-03-16%20ESG%20Investor%20Update%20Webinar_Final.pdf (updated Mar 2023; viewed Mar 2024).
44. Merck and Co. Our Commitment to ESG: Performance and Progress. Rahway, NJ, USA: Merck & Co, 2022. https://www.msd.com/wp-content/uploads/sites/9/2022/05/ESG-Fact-Sheet-MSD.pdf (viewed March 2024)
45. Merck & Co. Environmental, social & governance (ESG) progress report 2021/2022. Rahway: Merck & Co, 2022. https://www.msd.com/wp-content/uploads/sites/9/2022/08/MSD-ESG-Report-21-22.pdf (viewed Mar 2024).
46. Merck & Co. Sustainability bond allocation report. Rahway: Merck & Co, 2023. https://www.merck.com/wp-content/uploads/sites/124/2023/12/2023-Sustainability-Bond-Report-Final.pdf (viewed Mar 2024).
47. Merck & Co. Sustainability financing framework. Rahway: Merck & Co, 2021. https://www.merck.com/wp-content/uploads/sites/124/2021/12/Merck-Sustainability-Financing-Framework.pdf (viewed Mar 2024).
48. Merck & Co. Impact report 2022/2023. Rahway: Merck & Co, 2023 https://www.msd.com/wp-content/uploads/sites/9/2023/08/MSD-ImpactReport_22-23.pdf (viewed Mar 2024).
49. Merck & Co. Public policy statement climate change. Rahway: Merck & Co, 2022. https://www.msd.com/wp-content/uploads/sites/9/2023/02/PUBLIC-CLIMATE-CHANGE-POLICY-JULY-2022-MSD.pdf (viewed Mar 2024).
50. Merck & Co. Climate change - policy statement. Rahway: Merck & Co, 2023. https://www.merck.com/wp-content/uploads/sites/124/2020/04/Climate-change_MRK_DEC23.pdf (viewed Mar 2024).
51. Merck & Co. Merck accelerates climate goals; announces carbon neutrality in operations by 2025. Rahway: Merck & Co, 2021. https://s2.q4cdn.com/584635680/files/doc_news/Merck-Accelerates-Climate-Goals-Announces-Carbon-Neutrality-in-Operations-by-2025-2021.pdf (updated Apr 2021; viewed Mar 2024).
52. AstraZeneca. Ambition zero carbon. Cambridge: AstraZeneca, 2023. https://www.astrazeneca.com/content/dam/az/Sustainability/2023/pdf/Ambition-Zero-Carbon.pdf (viewed Mar 2024).

53. AstraZeneca. What science can do: AstraZeneca task force on climate-related financial sisclosures statement. Cambridge: AstraZeneca, 2022. https://www.astrazeneca.com/content/dam/az/Sustainability/2023/pdf/AstraZeneca_TCFD_Report_2022.pdf (viewed Mar 2024).
54. AstraZeneca. AstraZeneca advances UK clean heat and energy efficiencies with £100m commitment. Cambridge: AstraZenenca, 2023. https://www.astrazeneca.com/content/dam/az/media-centre-docs/article_files/articles2023/press-release-UK-az-clean-heat-announcement-sept-2023.pdf (updated Sep 2023; viewed Mar 2024).
55. AstraZeneca. What science can do: AstraZeneca annual report and form 20-F information 2023. Cambridge: AstraZeneca, 2024. https://www.astrazeneca.com/content/dam/az/Investor_Relations/annual-report-2023/pdf/AstraZeneca_AR_2023.pdf (viewed Mar 2024).
56. AstraZeneca. Sustainability report 2023. Cambridge: AstraZeneca, 2024. https://www.astrazeneca.com/content/dam/az/Sustainability/2024/pdf/Sustainability-Report-2023.pdf (viewed Mar 2024).
57. AstraZeneca. Investment case. Cambridge: AstraZeneca, 2023. https://www.astrazeneca.com/content/dam/az/Investor_Relations/events/AZN-investment-case.pdf (updated Jun 2023; viewed Mar 2024).
58. AstraZeneca. Full year and Q4 2023 results announcement. Cambridge: AstaZeneca, 2023. https://www.astrazeneca.com/content/dam/az/PDF/2023/fy/Full-year-and-Q4-2023-results-announcement.pdf (updated Feb 2024; viewed Mar 2024).
59. Vertex. Corporate responsibility report. Boston: Vertex, 2023. https://www.vrtx.com/sites/global/files/2022_Vertex_Corporate_Responsibility_Report.pdf (viewed Mar 2024).
60. Vertex. Vertex's commitment to health, safety and sustainability. Boston: Vertex, 2021. https://www.vrtx.com/sites/global/files/Vertex-Sustainability-Health-Safety-Commitment.pdf (viewed Mar 2024).
61. Viatris. Building sustainable access to medicine - 2022 sustainability report. Canonsburg: Viatris, 2023. https://www.viatris.com/-/media/project/common/viatris/csrpromo/pdfs/2023_345_2023csrreport_revised.pdf (viewed Mar 2024).
62. Viatris. Supplier code of conduct. Canonsburg: Viatris, 2022. https://www.viatris.com/-/media/project/common/viatris/pdf/corporate-governance/supplier-code-of-conduct.pdf (viewed Mar 2024).
63. Viatris. Proxy statement - annual meeting of shareholders. Canonsburg: Viatris, 2023. https://investor.viatris.com/static-files/95ab4f44-20c2-43ca-a433-f63ff296e5d3 (viewed Mar 2024).
64. Viatris. How is Viatris addressing climate change? Canonsburg: Viatris, 2023. https://www.viatris.com/-/media/project/common/viatris/pdf/english/csr/faq_1_climate_change.pdf (viewed Mar 2024).
65. Garijo B, Hudson P, Rim J et al. Open letter on supplier targets from members of the sustainable markets initiative health systems task force. Sustainable Markets Inititive, 2023. https://assets.roche.com/f/109506/x/5388424c6e/joint-ceo-letter_final_200723.pdf?cv=1689688147120 (updated Jul 2023; viewed Mar 2024).
66. Roche. Climate risk management. Basel: Roche, 2023. https://assets.roche.com/f/176343/x/01d6bff2e2/climate-risk-management.pdf (viewed Mar 2024).
67. Roche. Our SHE goals and performance: energy consumption. Basel: Roche, 2023. https://assets.cwp.roche.com/f/176343/x/b894e8ad96/she-energy-consumption.pdf (viewed Mar 2024).
68. Roche. Environmental sustainability: ESG investor event. Basel: Roche, 2023. https://assets.roche.com/f/126832/x/ec07ff472d/ir-esg-event-2023.pdf (updated May 2023; viewed Mar 2024).
69. Roche. Statement: Roche signs up to the Science Based Targets Initiative and the Sustainable Markets Initiative to further deepen the commitment to climate actions. Basel: Roche, 2023. https://assets.roche.com/f/176343/x/aad0bd63e9/17112022_media_statement_sbti_smi.pdf (updated Nov 2023; viewed Mar 2024).
70. Roche. Our SHE goals and performance: greenhouse gas emissions. Basel: Roche, 2023. https://assets.roche.com/f/176343/x/17779cc711/greenhouse-gas-emissions.pdf (viewed Mar 2024).

71. Roche. Our SHE goals and performance: scope 3 greenhouse gas emissions. Basel: Roche, 2023. https://assets.roche.com/f/176343/x/2d01fee21e/scope-3-greenhouse-gas-emissions.pdf (viewed Mar 2024).
72. Roche. Annual report 2023. Basel: Roche, 2024. https://assets.roche.com/f/176343/x/98b8e2ba9d/ar23e.pdf (viewed Mar 2024).
73. Roche. Roche 2023 results. Basel: Roche, 2024. https://assets.roche.com/f/176343/x/ac48d3ba3b/irp240201-a.pdf (updated Feb 2024; viewed Mar 2024).
74. Roche. Active worldwide – at home in switzerland. Basel: Roche, 2023. https://assets.roche.com/f/164639/x/7c5e6a564b/roche-in-der-schweiz-2023-en.pdf (viewed Mar 2024).

STROBE Statement—Checklist of items that should be included in reports of *cross-sectional studies*. Note: The page numbers refer to the submitted manuscript, not to the published article or its supplementary file.

	**Item No**	**Recommendation**	**Page No**
**Title and abstract**	1	(*a*) Indicate the study's design with a commonly used term in the title or the abstract	1
		(*b*) Provide in the abstract an informative and balanced summary of what was done and what was found	1
**Introduction**			
Background/rationale	2	Explain the scientific background and rationale for the investigation being reported	2
Objectives	3	State specific objectives, including any prespecified hypotheses	2
**Methods**			
Study design	4	Present key elements of study design early in the paper	3
Setting	5	Describe the setting, locations, and relevant dates, including periods of recruitment, exposure, follow-up, and data collection	3
Participants	6	(*a*) Give the eligibility criteria, and the sources and methods of selection of participants	3 + PRISMA
Variables	7	Clearly define all outcomes, exposures, predictors, potential confounders, and effect modifiers. Give diagnostic criteria, if applicable	3
Data sources/ measurement	8*	For each variable of interest, give sources of data and details of methods of assessment (measurement). Describe comparability of assessment methods if there is more than one group	*3*
Bias	9	Describe any efforts to address potential sources of bias	4
Study size	10	Explain how the study size was arrived at	3
Quantitative variables	11	Explain how quantitative variables were handled in the analyses. If applicable, describe which groupings were chosen and why	4
Statistical methods	12	(*a*) Describe all statistical methods, including those used to control for confounding	4
		(*b*) Describe any methods used to examine subgroups and interactions	N/A
		(*c*) Explain how missing data were addressed	7
		(*d*) If applicable, describe analytical methods taking account of sampling strategy	4
		(*e*) Describe any sensitivity analyses	4
**Results**			
Participants	13*	(a) Report numbers of individuals at each stage of study—eg numbers potentially eligible, examined for eligibility,	3

		confirmed eligible, included in the study, completing follow-up, and analysed	
		(b) Give reasons for non-participation at each stage	N/A
		(c) Consider use of a flow diagram	Y
Descriptive data	14*	(a) Give characteristics of study participants (eg demographic, clinical, social) and information on exposures and potential confounders	3 + PRISMA
		(b) Indicate number of participants with missing data for each variable of interest	PRISMA
Outcome data	15*	Report numbers of outcome events or summary measures	4
Main results	16	(*a*) Give unadjusted estimates and, if applicable, confounder-adjusted estimates and their precision (eg, 95% confidence interval). Make clear which confounders were adjusted for and why they were included	N/A
		(*b*) Report category boundaries when continuous variables were categorized	N/A
		(*c*) If relevant, consider translating estimates of relative risk into absolute risk for a meaningful time period	N/A
Other analyses	17	Report other analyses done—eg analyses of subgroups and interactions, and sensitivity analyses	N/A
**Discussion**			
Key results	18	Summarise key results with reference to study objectives	5
Limitations	19	Discuss limitations of the study, taking into account sources of potential bias or imprecision. Discuss both direction and magnitude of any potential bias	7
Interpretation	20	Give a cautious overall interpretation of results considering objectives, limitations, multiplicity of analyses, results from similar studies, and other relevant evidence	7
Generalisability	21	Discuss the generalisability (external validity) of the study results	7
**Other information**			
Funding	22	Give the source of funding and the role of the funders for the present study and, if applicable, for the original study on which the present article is based	8

*Give information separately for exposed and unexposed groups.

Note: An Explanation and Elaboration article discusses each checklist item and gives methodological background and published examples of transparent reporting. The STROBE checklist is best used in conjunction with this article (freely available on the Web sites of PLoS Medicine at http://www.plosmedicine.org/, Annals of Internal Medicine at http://www.annals.org/, and Epidemiology at http://www.epidem.com/). Information on the STROBE Initiative is available at www.strobe-statement.org.
